# Experts Opinion

**DOI:** 10.5005/jp-journals-10008-1107

**Published:** 2012-08-16

**Authors:** Ivan Goldberg, Franz Grehn, Paul Chew

**Affiliations:** 1 Clinical Associate Professor, Department of Ophthalmology, Director of Glaucoma Services, Sydney Eye Hospital, Australia; 2 Chairman and Professor, Department of Ophthalmology, The University of Wurzburg, Germany; 3 Associate Professor, Department of Ophthalmology, Yong Loo Lin School of Medicine, National University of Singapore, Senior Consultant, Department of Ophthalmology, NUH, Singapore

## Abstract

In this editions’ ‘Experts Opinion’, the management of hy-potony and choroidal effusion following glaucoma filtration surgery is discussed. We are delighted to welcome Clinical Associate Professor Ivan Goldberg and Professor Franz Grehn and Paul Chew to the panel to provide opinion on this very challenging clinical situation.

## THE CASE

An 81-year-old female undergoes a left primary trabeculec-tomy with mitomycin C for progressing glaucoma despite maximum tolerated medical therapy. Postoperatively the bleb overdrains resulting in hypotony. Within 3 months of surgery, she has left vision of hand movements secondary to kissing choroidal effusions.

**A. What is your management protocol for hypotony following trabeculectomy?**

*Ivan Goldberg:* Defined simply as ‘low intraocular pressure’ (IOP), in the absence of an aqueous leak, hypotony in itself does not warrant treatment, as the majority of ‘overly soft’ eyes will show a slowly increasing IOP. If there are no other findings, I decrease the frequency of topical steroids and avoid 5-FU augmentation. If there is shallowing of the anterior chamber (AC) with or without shallow choroidal detachments, particularly in phakic eyes, cycloplegics are introduced if not already in use. Any evidence of progressive AC shallowing or increasing suprachoroidal fluid leads to progressive interventions: Large bandage soft CL to try to slow bleb flow at first and then a Simmons shell over such a CL.
  *Franz Grehn:* If hypotony (<5 mm Hg) occurs in the first postoperative days, confirmation of ‘risky hypotony’ can be ascertained by palpation (very soft or some tension). IOP measurements, even applanation tonometry is not very precise in the range of 1 to 5 mm Hg.

### Examination

1. Fundus examination by binocular indirect ophthalmos-copy to exclude or confirm choroidal detachment.
2. Biomicroscopy of the macula.
3. Ultrasound for choroidal swelling if there is no bullous choroidal detachment.
4. OCT of the macula to exclude macular edema.
5. Transconjunctival inspection of scleral flap sutures whether one has inadvertently opened.
6. Disinsertion of the scleral spur or ciliary body detachment is a very rare cause of hypotony. If this is suspected, UBM examination is needed to confirm ciliary body detachment, as the therapeutic approach is different (transscleral fixation).

### Therapy

1. If 1 to 5 is negative and IOP by palpation does not seem critical, the situation is closely followed without intervention.
2. If moderate choroidal swelling without bullae is present, follow closely. Injection of hyaluronic acid may be considered to produce a transient IOP elevation (0.25 ml).
3. If overfiltration seems critical and choroidal bullae appear, transconjunctival 10/0 nylon sutures (as proposed by Pfeiffer) are used to suture down the scleral flap, usually in the gaps between the original sutures (this does not result in external fistulation).
4. If the cause for overfiltration is a broken suture, open revision is preferred.
5. If choroidal bullae persist despite transconjunctival sutures, choroidal drainage is considered, and simultaneous open scleral revision with sutures is performed to reduce flow through the scleral flap (choroidal drainage without treatment of overfiltration usually leads to relapses).

### Other Approaches

1. Matress sutures (barrier sutures) or compression sutures to reduce the filtration area are rarely used as they are less effective.
2. Injection of autologous blood is not recommended as it usually is ineffective.

*Paul Chew:* In the immediate postoperative period, I will attempt to elicit scarring by decreasing the frequency of steroid eye drops. Visual acuity should be monitored closely with emphasis on posterior segment evaluation to detect hypotony maculopathy and choroidals. If after 3 weeks of waiting, hypotony persists and threatens vision, I will plan for bleb revision and repair to stitch the scleral flap tight thus restricting aqueous flow through sclerostomy. An amniotic membrane or pericardial patch graft overscleral flap may be used to totally seal the trabeculectomy by stitching or using glue overscleral flap. The patient must be well informed of the possibility of returning to topical antiglaucoma eye drops or a second operation (e.g. glaucoma drainage device GDD implantation) for IOP control.

**B. Discuss your management of choroidal effusion following trabeculectomy?**

*Ivan Goldberg:* A flat or almost flat AC as well as ‘kissing’ choroidals requires immediate intervention, which would consist of suprachoroidal fluid drainage and AC reformation with a high viscosity space maintainer such as Healon GV or Healon 5.
  Franz Grehn:
  1. Development of bullous choroidal detachment should be avoided in an early stage, as this situation usually leads to gross increase of uveoscleral outflow and underperfusion of the bleb, resulting in obliteration of the bleb by later scar formation. Hence, my policy is early intervention if signs of choroidal swelling appear, but wait and see at any IOP if these signs are absent.
  2. Transconjunctival sutures can be correctly placed, if the conjunctiva is transparent or is made transparent by a glass spatula. They can be removed easily if sutures are too tight and an elevated IOP results. However, these sutures melt through the conjunctiva within some days and become looser on their own. Conjunctival hyperemia should be closely followed and scar formation should be avoided, if signs of increased wound healing (sometimes caused by the sutures) appear. Deeply melted sutures can be lasered if needed.
  3. An injection of hylauronic acid alone rarely results in long-term stabilization but may be added to scleral sutures, if an IOP spike is considered helpful to flatten choroidal bullae.
  4. Choroidal drainage is often not effective if used as a stand-alone procedure. The cause of overfiltration must be ascertained and treated. Therefore, transscleral choroidal drainage is a rare procedure in my hands.
  5. In children and rarely in adults scleral sutures may cheese-wire through the thin scleral flap and over-filtration will occur through the holes of the stiches (when done with a spatula needle). In these cases, an open revision deems necessary: Remove those stiches that create fistulizing holes and replace sutures using a round needle with 10/0 nylon.
The patient underwent inferomedial deep sclerotomy resulting in almost complete resolution of the choroidal effusions. However, within 3 weeks, choroidal effusions had returned to predrainage levels. A second drainage via inferomedial and inferolateral deep sclerotomies was performed. Two months following this, the IOP remains low from over filtration and peripheral choroidal effusions are accumulating again.
  *Paul Chew:* For choroidal effusion not threatening the macula, I opt to be conservative and just wait for it to resolve which usually happens in most cases. For non-resolving choroidal effusion, kissing or not, lasting for 2 months and blocking the visual axis or causing macula/ retinal folds, I will consider drainage of the effusion.

**C. Please discuss the management of recurrent choroidal effusions in the setting of hypotony. At what stage should an overfiltering bleb be corrected and what technique/s would you use?**

*Ivan Goldberg:* Should this situation recur, such intervention might need to be repeated and in this case, transconjunctival scleral trapdoor reinforcement sutures might be necessary.
  If this intervention does not prevent reaccumulation of suprachoroidal fluid, formal trabeculectomy revision might well be required as one would suspect flap failure. In this situation, one would need to be prepared to reinforce the scleral trapdoor with donor cornea or sclera.
  *Franz Grehn:* As already stated, overfiltration should be corrected at any early stage of choroidal swelling (even if only ultrasound signs of choroidal swelling without bullae are present, but progressing). The rationale of this approach is the fact that blebs can scar down, if not perfused during excessive uveoscleral outflow when choroidal swelling and bullae are present. Techniques see above under a therapy.
  *Paul Chew:* The rare possibility of choroidal effusion syndrome should be considered and it may be useful to measure the posterior sclera for thickening that may compress the exit points of vortex veins. Localized zones of posterior scleritis that may also bring about recurrent choroidal effusions must be ruled out by performing B-scan ultrasonography. The traditional approach for posterior scleral thickening is partial sclerectomy around the zones of vortex vein but this is seldom needed these days. It is also prudent to investigate the status of ciliary body for any displacement or cyclodialysis cleft by ultrasound biomicroscopy, as such scenario may also give rise to chronic hypotony that may lead to phthisis bulbi. If any of these conditions is found, appropriate treatment will be instituted for resolution of ocular pathology (e.g. surgical closure of cyclodialysis cleft).
An overfiltering bleb that endangers vision due to hypotony maculopathy should be corrected surgically after initial measures to promote scarring around the bleb have been employed (e.g. decreasing or stopping anti-inflammatory eye drops or autologous blood injection of the bleb). Compression sutures to reduce the size of the bleb are a useful option if initial measures fail.

**D. In the 3 to 6 months following primary trabeculectomy, cataract in the operated eye has significantly progressed. When is it appropriate to perform cataract surgery posttrabeculectomy? Should technique be altered or any precautions taken?**

*Ivan Goldberg:* The longer the interval between successful trabeculectomy and subsequent cataract surgery, the less likely is the phacoemulsification to interfere with bleb function. If at all possible, 6 months from the most recent drainage surgery would be the goal. Postcataract surgery subconjunctival 5-FU should be considered depending on bleb inflammation and IOP levels. Topical steroids should be more intense than usual.
  Franz Grehn:
  1. Clear cornea phacoemulsification from a small temporal incision is the preferred technique, all cor-neoscleral approaches should be avoided.
  2. Even clear cornea cataract surgery bears the risk of obliteration of a previously functioning filtering bleb (20-30% bleb failure). Therefore, meticulous follow-up is mandatory. If IOP increase occurs shortly after cataract surgery, 5-FU injections and/or needling should be considered. In contrast to nonoperated eyes, the IOP rarely decreases after cataract surgery in eyes with previous successful filters.
*Paul Chew:* The best timing for cataract surgery after primary trabeculectomy will depend on how immediate is the need for visual rehabilitation, the visual potential of the eye and of course, the status of trabeculectomy bleb. It is a fact that cataract surgery will increase the risk of trabeculectomy failure and this risk is heightened by shorter interval between the 2 operations.

In the case of this patient, a visually significant cataract may be operated at this time after all the risks and benefits of the procedure have been explained and understood by the patient especially the need to control glaucoma with medical therapy or GDD surgery in the event of hastened trabeculectomy failure after cataract surgery. The surgeon should be prepared to deal with anterior chamber instability during phacoemulsification due to altered fluid dynamics resulting from the trabeculectomy surgery.

**E. In the meantime, glaucoma in the right eye remains uncontrolled. How would you manage this eye if:**

1. The CDR is 0.9 with advanced field loss threatening fixation
2. The CDR is 0.7 with mild field loss only
3. The patient is 60 years old

*Ivan Goldberg:* The responses to these three scenarios are linked: More advanced damage and younger age increase the stakes and the urgency of accelerating hypotensive therapy. As the surgical approach for the first eye (conventional augmented trabeculectomy) has proven so disappointing for patient and doctor, an alternative approach should be considered, with reduced risk of hypotony. Vide infra.
  Franz Grehn:

1. CDR 0.9 and threatened fixation: If IOP and field loss make an operation in the second (better) eye necessary, I would postpone this operation using MMT (without systemic CAI to avoid reduced aqueous flow through the filering bleb) and/or laser trabeculoplasty. With threatened fixation I would probably prefer canaloplasty if this is the better eye.
2. CDR 0.7, mild field loss: Remain conservative until progression occurs. MMT plus LTP, CPC if needed.
3. Age 60: Depending on visual field loss and progression, a 60-year-old glaucoma patient needs thorough long-term IOP control as he can become blind over his life-span. On the contrary, inventions are not urgent and decisions can be postponed until the first operated eye is safe and IOP controlled without medication.

*Paul Chew:* For the 3 situations listed above, I will suggest a combined trabeculectomy with MMC and cataract surgery (phacoemulsification with intraocular lens insertion). Coexisting cataract is a usual finding in patients with glaucoma so addressing the two conditions at the same time may be a valid option. The course of action, whether to do combined procedures or one procedure at a time will depend not only on patient factors but also of equal importance is the surgeon’s expertise and comfort.

**F. How do you overcome the patients reluctance/refusal to undergo surgery to her ‘good eye’ given the complications with the other eye?**

*Ivan Goldberg:* I had offer her an alternative surgical approach, such as nonpenetrating glaucoma drainage surgery (by referring her if necessary to a colleague with the requisite experience) or a so-called minimally invasive technique, such as an iStent. In my city, this latter approach would require considerable effort and time to obtain access to the device.
  *Franz Grehn:* Depending on the visual field damage and progression I would optimize medical therapy, possibly using laser (LTP or CPC). If surgery is needed, I would offer canaloplasty.
  Generally, in decisions on glaucoma surgery the patient’s confidence and acceptance is a major factor to perform the various therapeutical measures in the postoperative period.
  *Paul Chew:* Patient’s awareness and full understanding of his current situation and its impact/significance to prognosis of the disease may help ease anxiety and reluctance to accept suggested management. Once the patient is well informed, then he is free to make a wise decision regarding his care. In the end, I will respect whatever is the patient’s choice.

